# Large Deviations Properties of Maximum Entropy Markov Chains from Spike Trains

**DOI:** 10.3390/e20080573

**Published:** 2018-08-03

**Authors:** Rodrigo Cofré, Cesar Maldonado, Fernando Rosas

**Affiliations:** 1 Centro de Investigación y Modelamiento de Fenómenos Aleatorios, Facultad de Ingeniería, Universidad de Valparaíso, Valparaíso 2340000, Chile; 2 IPICYT/División de Matemáticas Aplicadas, Instituto Potosino de Investigación Científica y Tecnológica, San Luis Potosí 78216, Mexico; 3 Centre of Complexity Science and Department of Mathematics, Imperial College London, London SW7 2AZ, UK; 4 Department of Electrical and Electronic Engineering, Imperial College London, London SW7 2AZ, UK

**Keywords:** computational neuroscience, spike train statistics, maximum entropy principle, large deviation theory, out-of-equilibrium statistical mechanics, thermodynamic formalism, entropy production

## Abstract

We consider the maximum entropy Markov chain inference approach to characterize the collective statistics of neuronal spike trains, focusing on the statistical properties of the inferred model. To find the maximum entropy Markov chain, we use the thermodynamic formalism, which provides insightful connections with statistical physics and thermodynamics from which large deviations properties arise naturally. We provide an accessible introduction to the maximum entropy Markov chain inference problem and large deviations theory to the community of computational neuroscience, avoiding some technicalities while preserving the core ideas and intuitions. We review large deviations techniques useful in spike train statistics to describe properties of accuracy and convergence in terms of sampling size. We use these results to study the statistical fluctuation of correlations, distinguishability, and irreversibility of maximum entropy Markov chains. We illustrate these applications using simple examples where the large deviation rate function is explicitly obtained for maximum entropy models of relevance in this field.

## 1. Introduction

Spiking neuronal networks are perhaps the most sophisticated information processing devices that are available for scientific inquiry. There exists already an understanding of their basic mechanisms and functionality: they are composed by interconnected neurons which fire action potentials (also known as “spikes”) collectively in order to accomplish specific tasks, e.g., sensory information processing or motor control [1]. However, the interdependencies in the spiking activity of populations of neurons can be extremely complex. In effect, these interdependencies can involve neighboring or also distant cells, being established either via structural connections, i.e., physical mediums such as synapses, or by functional connections reflected through spike correlations [2].

Understanding the way in which neuronal networks process information requires disentangling structural and functional connections while clarifying their interplay, which is a challenging but critical issue [3,4]. For this aim, networks of spiking neurons are usually measured using in vitro or in vivo multi-electrode-arrays, which connect neurons to electronic sensors specially designed for spike detection. Recent progress in acquisition techniques allows the simultaneous measurement of the spiking activity from increasingly large populations of neurons, enabling the collection of large amounts of experimental data [5]. Prominent examples of spike train recordings have been obtained from vertebrate retina (salamander, rabbit, and degus) [6,7,8] and cat cortex [9].

However, despite the progress in multi-electrode and neuroimaging recording techniques, modeling the collective spike train statistics is still one of the key open challenges in computational neuroscience. Analysis over recorded data has shown that, although the neuronal activity is highly variable (even when presented repeatedly the same stimulus), the statistics of the response is highly structured [10,11]. Therefore, it seems that much of the inner dynamics of neuronal networks is encoded in the statistical structure of the spikes. Unfortunately, traditional methods of estimation, inference, and model selection are not well-suited for this scenario since the number of possible binary patterns that a neuronal network can adopt grows exponentially with the size of the population. In fact, even long experimental recordings usually contain only a small subset of the possible spiking patterns, which makes the empirical frequencies poor estimators for the underlying probability distribution. For practical purposes, this induces dramatic limitations, as standard inference tools become unreliable as soon as the number of considered neurons grows beyond 10 [6].

Given the binary nature of the spiking data, it is natural to relate neuronal networks and digital communication system via Shannon’s information theory. A possibly more subtle way of establishing this link is provided by the physics literature that studies stochastic spins systems. In fact, a succession of research efforts has helped develop a framework to study the spike train statistics based on tools of statistical physics, namely the maximum entropy principle (MEP), which provides an intuitive and tractable procedure to build a statistical model for the whole neuronal network. In 2006, Schneidman et al. [6] and Pillow et al. [12] used the MEP to characterize the spike train statistics of the vertebrate retina responding to natural stimuli, constraining only range one features, namely firing rates and instantaneous pairwise interactions. Since then, the MEP approach has become a standard tool to build probability measures in the field of spike train statistics [6,8,12,13]. This approach has triggered fruitful analyses of the neural code, including works about criticality [14], redundancy and error correction [7] among other intriguing and promising topics.

Although relatively successful, this approach for linking neuronal populations and statistical mechanics is based on assumptions that go against fundamental biological knowledge. Firstly, these works assume that the spike patterns are statistically independent of past and future activities of the network. In fact, and not surprisingly, there exists strong evidence supporting the fact that memory effects play a major role in spike train statistics [8,9,15]. Secondly, most studies that apply statistical mechanics to analyze neuronal data use tools that assume that the underlying system is in thermodynamic equilibrium. However, it has been recognized that being out-of-equilibrium is one of the distinctive properties of living systems [16,17,18]. Consequently, any statistical description that is consistent with the out-of-equilibrium condition of living neuronal networks should reflect some degree of time asymmetry (i.e., time irreversibility), which can be characterized using Markov chains [19,20,21,22,23,24].

As a way of addressing the above observations, some recent publications study maximum entropy Markov chains (MEMC) based on a variational principle from the thermodynamic formalism of dynamical systems (see for instance [8,24,25]). This framework is an extension of the classic approach based on the MEP that considers correlation of spikes among neurons simultaneously and with different time delays as constraints, being able in this way to account for various memory effects.

Most of the literature in spike train statistics via the MEP pays little attention to the fact that model estimation is done based on finite data (errors due to statistical fluctuations are likely to occur in this context). As the MEP can be seen as a statistical inference procedure, it is natural to inquire about the uncertainty (i.e., fluctuations and convergence properties) related to the inferred MEMC, or, in other words, ask for the robustness of the inference as a function of the sampling size of the underlying data set. Quantifying this error is particularly relevant in the light of recent results that suggest that the parameters inferred by the MEP approach in the context of experimental biological recordings are sharply poised at criticality [7,26]. On the other hand, once the MEMC has been inferred, it is also important to quantify how well a sample of the MEMC reproduce the average values of features of interest and how likely is that a sample of the MEMC produce a “rare” or unlikely event.

To provide some first steps in addressing the above issues, this paper studies the MEMC framework using tools from large deviation theory (LDT) [27,28]. We exploit the fact that the average values of features obtained from samples of the MEMC satisfy a large deviation property, and use LDT techniques to estimate their fluctuations in terms of the sampling size. We also show how to compute the rate functions using the tilted transition matrix technique and the Gärtner–Ellis theorem. It is to be noted that there is a large body of theoretical work linking the maximum entropy principle and large deviations [27,29]. However, these techniques have been scarcely used in spike train analysis (only to study the i.i.d case [30,31,32,33]), most likely because of the lack of a suitable introduction of these concepts within the neuroscientific literature. It is our hope that these applications might trigger the interest of the computational neuroscience community into the large deviation literature. Consequently, another goal of this paper is to provide an accessible introduction of the MEMC and LDT formalisms to the community of computational neuroscience, avoiding some technicalities while preserving the core ideas and intuitions. To the best of our knowledge, this manuscript presents the first attempt to bring these two topics together in the context of spike train statistics. This article is part of a more ambitious program that attempts to build a more unified theoretical structure and a complete toolbox helpful to approach spike train statistics using the thermodynamic formalism [24,25].

The rest of this paper is organized as follows. Section 2 presents the basic definitions and tools needed to apply large deviations techniques further in the paper. In particular, we present the maximum entropy principle framed in the thermodynamic formalism as a variational principle. In Section 3, we introduce some basic aspects of the theory of large deviations. In Section 4, we focus on the empirical averages of features. We present some examples of relevance in spike train statistics, where we are able to compute explicitly the rate function for each feature in the maximum entropy potential. In Section 5, we present further applications of the theory of large deviations in this field with a list of illustrative examples and finally we present our conclusions in Section 6.

## 2. Preliminaries

This section introduces the general definitions, notations and conventions that are used throughout the paper, providing in turn the necessary background for the unfamiliar reader.

### 2.1. Data Binarization and Spike Trains

Let us consider a set of measurements from a network of *N* interacting neurons. The “raw data” consist of *N* continuous signals containing the extra-cellular potential (electrical potential measured outside of the cell) of each of the neurons recorded over the length of the experiment. These data are processed by spike sorting algorithms [34,35], which are signal processing techniques designed to extract the spiking activity of each neuron.

**Figure 1:**
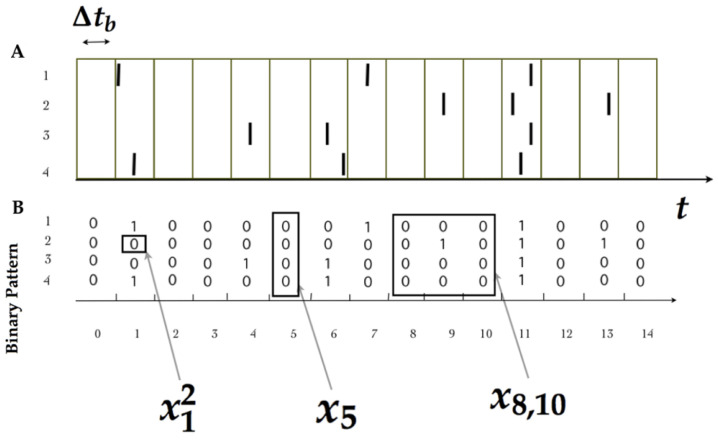
(**A**) Each bar indicates a spike of a neuron indexed from 1 to 4 in continuous time. (**B**) After binning $\Delta t_{b}$, the spiking activity is transformed into binary patterns in discrete time. We illustrate the notation used throughout this paper.

Neurons have a minimal characteristic time in which no two spikes can occur, called “refractory period” [36], which provides a natural time-scale that can be used for “binning” (i.e., for discretizing) the time index of the measurements, denoted by $\Delta t_{b}$ (When binning, sometimes it can be useful to go beyond the refractory period. In those cases, two spikes may occur within the same time bin. The convention is to consider this event equivalent to just one spike.). Denoting the time index by the integer variable *t*, one can say that $x_{t}^{k}=1$ whenever the *k*-th neuron emits a spike during the *t*-th time bin, while $x_{t}^{k}=0$ otherwise. This standard procedure transforms experimental data into sequences of binary patterns (see Figure 1).

A *spike pattern* is the spike-state of all the measured neurons at time bin *t*, which is denoted by $x_{t}:={\left[x_{t}^{k}\right]}_{k=1}^{N}$. A *spike block* is a consecutive sequence of spike patterns, denoted by $x_{t,r}:={\left[x_{s}\right]}_{s=t}^{r}$. While the length of the spike block $x_{t,r}$ is r−t+1, is also useful to consider spike blocks of infinite length starting from time t=0, which are denoted by x. Finally, is this paper, we consider that a *spike train* is either a spike block of finite length or an infinite sequence of spiking patterns, which will be useful later when discussing asymptotic properties. The set of all possible spike blocks of length *R* corresponding to a network of *N* neurons is denoted by $A_{R}^{N}$. The set of all spike blocks of infinite length is denoted by $\Omega \equiv A_{N}^{N}$, which is a useful mathematical object as clarified below. Let us define $proj_{R}:\Omega \to A_{R}^{N}$ the natural projection given by $proj_{R}\left(x\right)=x_{0,R-1}$.

### 2.2. Features

Following the machine-learning nomenclature, a *feature* is a function that extracts a property of interest from the data. Formally, is defined as a function f:Ω→R that associates a real number to each x∈Ω. The feature *f* is said to have a temporal range or simply a range *R* if for every x,y∈Ω such that x≠y, one has that f(x)=f(y) if and only if $x_{0,R-1}=y_{0,R-1}$, that is, if *f* only depends on the first *R* spike patterns of the spike-train. A special class of features, over which this work is focused on, are binary functions consisting of finite products of spike states, i.e.,

$$f_{l}\left(x\right)=\prod_{k=1}^{q}x_{t_{k}}^{i_{k}}.$$

Above, *l* is a shorthand notation for the set ${\left\{t_{k},i_{k}\right\}}_{k=1}^{q}$, where ${\left[t_{k}\right]}_{k=1}^{q}$ and ${\left[i_{k}\right]}_{k=1}^{q}$ are collections of time and neuron indexes, respectively, and *q* is the number of spikes considered by the feature. Correspondingly, for a given index *l*, one has $f_{l}\left(x\right)=1$ if and only if the $i_{k}$-th neuron spikes at time $t_{k},$ for all k∈{1,…,q} in the spike-train x, while $f_{l}\left(x\right)=0$ otherwise. Note that, when considering features of range R≥1, the firing times $t_{k}$ are constrained within the interval $\left\{\;0,\ldots ,R-1\;\right\}$. We define the reduced feature $\tilde{f}:A_{N}^{R}\to R$ such that

$$\tilde{f}\left(x_{0,R-1}\right)=\tilde{f}\left(proj_{R}\left(x\right)\right)=f\left(x\right).$$

### 2.3. Statistical Structure

For a given spiking neuronal network involved in a particular experimental protocol, the measured activity usually contains a significant amount of stochasticity that is characteristic of measurements at this spatiotemporal scale. This randomness is caused mostly by:

(i) the random variation in the ionic flux of charges crossing the cellular membrane per unit time at the post synaptic button due to the binding of neurotransmitter;
(ii) the fluctuations in the current resulting from the large number of opening and closing of ion channels [37,38];
(iii) noise coming from electrical synapses [39].

To capture this stochasticity within our modeling, it is natural to endow Ω with a probabilistic structure. For this, we assume there exists a probability distribution p{·} over Ω that quantifies the intrinsic randomness that is associated to the spiking phenomena. From this point of view, all A⊂Ω are events that might take place with probability p{A}. Following a standard practice in computational neuroscience, we assume that the stochastic process generating the spikes is stationary, i.e., that their statistics do not change in time. As we discuss below, this assumption is crucial for the maximum entropy inference. Although an extension of our approach to a non-stationary scenario is possible, we focus here on the stationary case as it greatly simplifies the presentation. Using the stationary assumption, given the probability distribution of the whole process p{·} one can define a unique corresponding probability distribution over $A_{R}^{N}$ following the natural projection, given by:

(1) $$p_{R}\left\{B\in A_{R}^{N}\right\}:=p\left\{proj_{R}^{-1}\left(B\right)\in \Omega \right\}.$$

As a consequence of assuming a stochastic process guiding the neuronal activity, a feature f:Ω→R becomes a random variable. Consequently, the statistics of *f* are defined by

p{f=a}=p{x∈Ω|f(x)=a}.

In particular, considering the feature $f\left(x\right)=x_{t}^{k}$, one can note that individual spike-states (as well as spike patterns and spike blocks) become discrete random variables. As a convention, we denote $X_{t}^{k}$ a random spike-state that follows an implicit underlying probability distribution p{·}, while lower-case expressions (e.g., $x_{t}^{k}$) are used for denoting concrete realization of these random variables. The mean value of a feature *f* with respect to the probability p{·} is given by:

$$E_{p}\left\{f\right\}=\sum_{x\in \Omega }f\left(x\right)p\left\{x\right\}.$$

For the case of features of range *R*, the mean value can be expressed alternatively as:

$$E_{p}\left\{f\right\}=\sum_{x_{0,R-1}\in A_{R}^{N}}\tilde{f}\left(x_{0,R-1}\right)p_{R}\left\{x_{0,R-1}\right\}=E_{p_{R}}\left\{\tilde{f}\right\}$$

which is a finite sum. Above, $\tilde{f}$ is the reduced feature, as defined in Section 2.2.

### 2.4. Empirical Averages

Let us consider spiking data of the form $x_{0,T-1}$, where *T* is the sample length. Although in general the underlying probability measure p{·} that governs the spiking activity is unknown, it is useful to use the available data to estimate the mean values of specific features. If *f* is a feature of range *R*, the empirical average value of *f* from the sample $x_{0,T-1}$ is

(2) $$A_{T}\left(f\right)=\frac{1}{T-R+1}\sum_{i=0}^{T-R}f\left(x_{i,R-1+i}\right).$$

In particular, for features of range one, the previous expression becomes $A_{T}\left(f\right)=\frac{1}{T}\sum_{i=0}^{T-1}f\left(x_{i}\right)$.

An interesting questions is under which conditions $A_{T}\left(f\right)\to E_{p}\left\{f\right\}$ as *T* grows. This, and other convergence issues, are explored in Section 4.

## 3. Inference of the Statistical Model with the MEP

Following Section 2.3, the probability measure p{·} represents the inherent stochasticity of the spiking neural population under consideration. As p{·} is unknown, one would like to infer it from data. In the sequel, we first introduce the general MEP as a method for inferring p{·}. Then, we show this principle for the case where only synchronous constraints are considered. Finally, we present the case of where non-synchronous correlations are included to constrain the maximization problem.

### 3.1. Fundamentals of the MEP

The MEP was first proposed by E. T. Jaynes as a way for estimating probability distributions when the information for performing the inference is scarce [40]. Rooted in principles of statistical physics, this approach selects a probability measure that satisfies the evidence supported by the available information while leaving all other aspects as random as possible. For quantifying the corresponding randomness, Jaynes shows that the most natural metric is the Shannon entropy [41]. The probability measure found by this procedure is known as the *maximum entropy distribution*.

Formally, the MEP is a concave constrained maximization problem, where the constraints that define the optimization space correspond to the available information that guide the inference process. Accordingly, if additional constraints are introduced then the optimization space is reduced; this corresponds to the informative power of new information, which restricts the space of models that are consistent with it.

The inference procedure based on the MEP follows the following steps:

I. Choose *K* features of interest $f_{1},\ldots ,f_{K}$ (cf. Section 2.2).
II. Using the available data $x_{0,T-1}$, compute the empirical averange of each feature $A_{T}\left(f_{k}\right):=c_{k}$.
III. Assuming stationarity, define the space of statistical models $M(c_{1},\ldots ,c_{K})\subset M$ given by
  $$M\left(c_{1},\ldots ,c_{K}\right)=\left\{g\in M|\;E_{g}\left\{f_{1}\right\}=c_{1},\ldots ,E_{g}\left\{f_{K}\right\}=c_{K}\right\},$$
  where M is the set of probability measures and $M(c_{1},\ldots ,c_{K})$ is the family of probability measures that are consistent with the empirical mean values $c_{1},\ldots ,c_{K}$ obtained in Step II.
IV. Defining the entropy rate of the stochastic process as
  (3) $$S\left\{p\right\}=\lim_{t\to \infty }\frac{1}{t}\sum_{x_{0,t-1}\in A_{t}^{N}}p_{t}\left\{x_{0,t-1}\right\}\log\frac{1}{p_{t}\left\{x_{0,t-1}\right\}},$$
  find the maximum entropy process, characterized by the probability measure
  (4) $$\hat{p}=\underset{q\;\in \;M(c_{1},\ldots ,c_{k})}{arg max}S\left\{q\right\}.$$

Some small remarks are to be said about this procedure. One can think of this as a data-driven algorithm, whose input is the data $x_{0,T-1}$ and output is the maximum entropy measure $\hat{p}$. The first two steps of the process are known in the machine learning literature as “feature selection” and “feature extraction”, respectively (see, e.g., [42,43]). The goal of these steps is to reduce the dimensionality of the input for the subsequent stages to prevent the selected model to overfitting the data (i.e., to include in the model effects of noise and biases due to the finiteness of the data). Hence, what drives the model selection stages is not the whole data but the quantities $c_{1},\ldots ,c_{K}$, which define the space to be explored in Step IV.

Steps III and IV are known as “model selection”. According to the the machine learning jargon, these steps deliver a generative model, in the sense the obtained model can later be used to generate new data. In this sense, it is interesting that, although the data are finite, the entropy rate calculated in Step IV is computed over all spike blocks of all lengths *t*, which is possible due to the generative nature of the candidate models. The inputs for the model selection stages are not the entire data $x_{0,T-1}$ but only the values $c_{1},\ldots ,c_{K}$, which represent the knowledge obtained from the data that guides the search in the space of candidate models. Moreover, as these quantities represent all the available knowledge one has about the underlying stochastic process generating the spikes, one would like to select a model that reflect that information while making no further assumptions. By recalling the work made by Claude Shannon on the analysis of information sources (cf. [44] and references therein), one can interpret the entropy rate as a measure of how hard is to predict the realization of a stochastic process. This implies, in turn, that the maximum entropy measure within $M\{c_{1},\ldots ,c_{K}\}$ is the most random, i.e., unstructured, among those which satisfies the constraints $A_{T}\left(f_{1}\right)=c_{1},\ldots ,A_{T}\left(f_{K}\right)=c_{k}$. Although the framework presented above is general enough to encompass the cases considering synchronous and non-synchronous constraints [21], when considering features of range R>2 the problem go beyond the realm of equilibrium statistical mechanics. We present an alternative way general enough to deal with systems assuming non-synchronous constraints. In Section 3.2, we present the method for finding the maximum entropy measure when only synchronous features are selected, leaving for Section 3.3 the more general situation including non-synchronous constraints.

### 3.2. Time-Independent Constraints

Assuming only synchronous interactions is the equivalent to only considering features of range one (i.e., features that consider neurons at the same time index, cf. Section 2.2), which leads to restricting the candidate models to those in where the present state is statistically independent of past and future states. Moreover, by the assumption of stationarity, this leads to modeling the collective spiking activity as a sequence of i.i.d. random variables. The challenge, in this case, is to estimate the corresponding distribution as reliably as possible. A large portion of the literature of maximum entropy spike train statistics focuses on synchronous interactions between neurons, implicitly neglecting interactions across time. Although this assumption induces a strong simplification, the resulting models have proven to be rich in structure and can provide interesting results and insights about the neural code [6,12]. In the following, we recall how this problem can be addressed using the MEP.

As a consequence of the assumptions of temporal independence and stationarity, it can be shown that Equation (4) is reduced to

(5) $${\hat{p}}_{1}=\underset{q_{1}\;\in \;M_{1}\left(c_{1},\ldots ,c_{k}\right)}{arg max}\sum_{x_{0}\in A_{1}^{N}}q_{1}\left\{x_{0}\right\}\log\frac{1}{q_{1}\left\{x_{0}\right\}}$$

where $M_{1}\left(c_{1},\ldots ,c_{k}\right)$ corresponds to the set of distributions $q_{1}$ over $A_{1}^{N}$ (cf. range one projections in Equation (1)) such that the constraints $E_{q_{1}}\left\{f_{k}\right\}=c_{k}$ are satisfied for each k=1,…,K. Note that the above sum is over the $2^{N}$ possible spike patterns, being a simpler condition than Equation (4). In fact, following a simple argument based on Lagrange multipliers and the concavity of the entropy, it can be shown that the distribution ${\hat{p}}_{1}$ that solves Equation (5) is unique. Moreover, it is a Boltzmann–Gibbs distribution [41]:

(6) $${\hat{p}}_{1}\left\{x_{0}\right\}=\frac{e^{-H_{\beta }\left(x_{0}\right)}}{Z(\beta )}\;\forall x_{0}\in A_{1}^{N};\;Z\left(\beta \right)=\sum_{x_{0}\in A_{1}^{N}}e^{-H_{\beta }\left(x_{0}\right)},$$

where $H_{\beta }$ is referred as the *energy or potential* function

(7) $$H_{\beta }\left(x_{0}\right)=\sum_{k=1}^{K}\beta_{k}{\tilde{f}}_{k}\left(x_{0}\right),$$

where $\beta \in R^{K}$ is the vector of Lagrange multipliers. Following the statistical physics literature, Z(β) is called the *partition function*, whose logarithm is referred as *free energy*.

Conversely, from the uniqueness property of the maximum entropy distribution, one can conclude that there is only one Boltzmann–Gibbs distribution ${\hat{p}}_{1}$ that belongs to $M(c_{1},\ldots ,c_{K})$, being the only solution of Equation (5). Interestingly, this alternative approach is much easier to solve the original optimization problem (In particular, $M_{1}\left\{c_{1},\ldots ,c_{k}\right\}$ is not easy to parameterize and hence the application of standard techniques of convex optimization (e.g., gradient decent) is not straightforward.). In effect, one only needs to find the values of the parameter vector $\beta_{k}$ that reproduces the empirical average values $c_{1},\ldots ,c_{K}$. Moreover, it is known that, for any Boltzmann–Gibbs distribution $p_{1}$, the following holds:

(8) $$\frac{\partial \ln Z(\beta )}{\partial \beta_{k}}=E_{{\hat{p}}_{1}}\left({\tilde{f}}_{k}\right).$$

Therefore, using Equation (8), one could find the appropriate values of β for which $E_{{\hat{p}}_{1}}\left\{{\tilde{f}}_{k}\right\}=c_{k}$ are satisfied (However, for practical purposes, this problem cannot be solved for systems with N>20 [8], so alternative procedures are needed. For the interested reader, we refer to [7,14,45,46]).

### 3.3. Non-Synchronous Constraints

A generalization of the previous approach is to include average values of features corresponding to interactions in the spiking activity across time as constraints. This is a natural assumption in biological spiking networks as it is expected that the spike of one neuron influence other subsequent spikes.

Statistical models with time-dependencies can be generated with the MEP by introducing features that involve different time indexes. In effect, selecting features of range *R* induces interdependencies and a corresponding “memory” in the model of length R−1, and thus it is natural to look for the best suited Markov chain over the state space $A_{N}^{R}$. A Markov chain model would allow expressing the probability of a spike train $x_{0,T}$ for T>R as

$$p\left\{x_{0,T}\right\}=\pi \left\{x_{0,R-1}\right\}P\left\{x_{1,R}|x_{0,R-1}\right\}\cdots P\left\{x_{T-R,T-1}|x_{T-R+1,T}\right\},$$

where *P* is a transition probability matrix (note that P{·,·} has a consistency requirement: for $w,y\in A_{N}^{R},P\left\{w|y\right\}>0$ only when $y_{1,R-1}=w_{0,R-2}$) and π is a corresponding invariant probability distribution (which is unique due to the ergodicity assumption, cf. Section 3.3.1) associated to *P*. Note that, due to the stationarity condition, the transition probabilities P{·|·} are constant over the realization of the whole process (see Appendix A for more details.).

Following the MEP, as described in Section 3.1, we look for a procedure for finding a Markov transition matrix *P* that maximizes its entropy rate while satisfying some constrains given the empirical averages of observables $f_{1},\ldots ,f_{K}$. For ergodic Markov chains, a well-known calculation (that can be found, e.g., in [44]) shows that the entropy rate, as given by Equation (3), is equivalent to the following simple expression:

(9) $$S_{KS}\left(\pi ,P\right)=-\sum_{i,j\in A_{N}^{R}}\pi_{i}\sum_{j}P_{ij}\log P_{ij}.$$

where $\pi_{i}=\pi \left\{x_{0,\;R-1}=i\right\}$ and $P_{ij}=P\left\{j|i\right\}$ for $i,j\in A_{R}^{N}$. It is important to notice that Equation (9) corresponds to the *Kolmogorov–Sinai entropy (KSE)* in the dynamical systems literature [47]. In general, Equation (9) is larger when, for a fixed *i*, the conditional probabilities $P_{ij}$ are closer to an uniform distribution, i.e., when knowing the transition statistics gives little certainty about the next step.

It can be shown that, if the considered features do not involve correlations across time (i.e., they are features of range one, cf. Section 2.2), then the resulting transition probabilities are such that the corresponding stochastic process is i.i.d (i.e., when $P_{ij}=\pi_{j}$). Interestingly, in this scenario, Equation (9) reduces to the Shannon entropy of $\pi_{i}$. This clarifies that this approach based on Markov chains extends the classical MEP and the results presented in Section 3.2.

Finding the MEMC raises, however, some extra technicalities with respect to the time-independent case. Recall that the goal is no longer to estimate a probability distribution, but to reconstruct from data a transition matrix *P* and a corresponding invariant measure π. On the one hand, the challenge is that as *P* and π are not independent parameters of the process (π has to be the eigenvector associated with the unitary eigenvalue of *P* [48]), and, on the other hand, although Lagrange multipliers method can still be applied for constraints of range two or more, the extension for non-synchronous constraints is not straightforward. For this reason, in the sequel, we explore an alternative route to build the MEMC based on the transfer matrix technique. This technique also provides further insightful connections with statistical physics and thermodynamics from which its large deviation properties arise naturally.

#### 3.3.1. Transfer Matrix Method

To find the MEMC associated with non-synchronous constraints, we follow the same ideas presented above in the time-independent case, but using different tools. We present them here.

Let us consider the set of features chosen to constrain the maximization of entropy rate (Step I in Section 3.1). We assume that the features chosen have a finite maximum range *R*. From these features, one can build the energy function $H_{\beta }$ (Equation (7)) of finite range *R* as a linear combination of these features. Using this energy function, we build a matrix denoted by $L_{H_{\beta }}$, so that for every $y,w\in A_{R}^{N}$ its entries are given as follows:

(10) $$L_{H_{\beta }}\left(y,w\right)=\left\{\begin{array}{cc} e^{H_{\beta }\left(yw_{R-1}\right)}\; & \mathrm{if}\;y_{1,R-1}=w_{0,R-2}\\ 0, & \mathrm{otherwise}. \end{array}\right.$$

By $yw_{R-1}$ we mean the word obtained by concatenation of $y_{1}$ and $w_{1,R-1}$. In the particular case of energy functions associated to range one features, the above matrix is defined as $L_{H_{\beta }}\left(y,w\right)=e^{H_{\beta }\left(y\right)}$. Assuming $H_{\beta }>-\infty$, the elements of the matrix $L_{H_{\beta }}$ are non-negative, which in turn implies ergodicity. Moreover, the matrix is primitive by construction, thus it satisfies the Perron–Frobenius theorem [49]. Hereafter, $L_{H}$ will be referred as the Ruelle–Perron–Frobenius matrix (RPF). Let us denote be ρ the largest eigenvalue of $L_{H}$, which because it satisfies the Perron–Frobenius theorem is an eigenvalue of multiplicity one and strictly larger in modulus than the rest of the eigenvalues [49]. We denote by *U* and *V* left and right eigenvectors of $L_{H_{\beta }}$ corresponding to the eigenvalue ρ. Notice that $U_{i}>0$ and $V_{i}>0$, for all $i\in A_{R}^{N}$.

**Figure 2:**
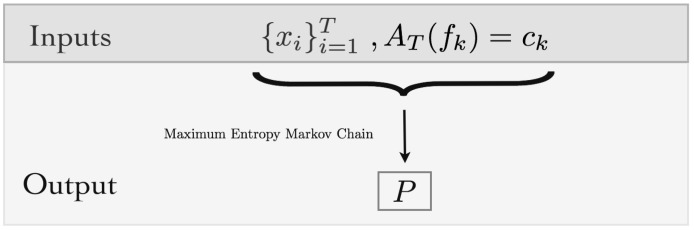
Algorithmic view of the maximum entropy Markov chains (MEMC): Inputs are the spike trains ${\left\{x_{i}\right\}}_{i=1}^{T}$ and the average values of a set of features. The output is the MEMC transition matrix *P*.

The RPF matrix is not the Markov matrix we are looking for, moreover, is not a stochastic matrix, but can be converted into a stochastic matrix. We recall that for an irreducible matrix *M* with spectral radius λ, and positive right eigenvector v associated to λ, then the *stochasticization* of *M* is the following stochastic matrix:

(11) $$S\left(M\right)=\frac{1}{\lambda }D^{-1}MD\;,$$

where *D* is the diagonal matrix with diagonal entries $D\left(i,i\right)=v_{i}$. Thus, in our context, the MEMC transition matrix is given as follows:

(12) $$P=S(L_{H_{\beta }}),$$

which is the main output of the MEMC approach (see Figure 2). The unique stationary probability measure π associated to *P* is explicitly given by

(13) $$\pi_{i}:=\frac{U_{i}\;V_{i}}{\langle U,V\rangle },\;\forall i\in A_{R}^{N},$$

where 〈U,V〉 is the standard inner product in $R^{NR}$ (we refer the reader to [49] for details and proofs).

#### 3.3.2. Thermodynamic Formalism

In the previous section, we have shown how to obtain the transition matrix and the invariant measure of a Markov chain. However, we have not yet included the constraints (we have just used the features to build the energy function); in other words, we have not yet fit the parameters of the MEMC. To fit the maximum entropy parameters, let us introduce the following quantity,

(14) $$P\left[\;H_{\beta }\;\right]=\underset{q\in M_{st}}{\sup}\left\{S\left\{q\right\}\;+\;E_{q}\left\{H_{\beta }\right\}\right\}$$

where $M_{st}$ is the set of all stationary probability measures in $A_{N}^{R}$ and $E_{q}\left\{H_{\beta }\right\}=\sum_{k=1}^{K}\beta_{k}\;E_{q}\left\{f_{k}\right\}$ is the average value of $H_{\beta }$ with respect to *q*. Solving the optimization problem in Equation (14), one gets the Markov measure we are looking for. Indeed, one knows from the thermodynamical formalism (see [50]) that for our energy function $H_{\beta }$ of range R≥2, there exists an unique translation invariant (stationary) Markov measure *p* associated to $H_{\beta }$ for which one has the constant M>1 such that,

(15) $$M^{-1}\leq \frac{p\{x_{1,n}\}}{\exp(\sum_{k=1}^{n-R+1}H\left(x_{k,k+R-1}\right)-\left(n+R-1\right)P\left[H_{\beta }\right])}\leq M,$$

that attains the supremum in Equation (14), that is $S\left\{p\right\}\;+\;E_{p}\left\{H_{\beta }\right\}$.

The quantity $P[H_{\beta }]$ is called *topological pressure*, which plays the role of the free energy in the statistical mechanics. The measure *p*, as defined by Equation (15), is known as the Gibbs measure in the sense of Bowen. Note that one can show that MEMCs are particular cases of these measures, associated to finite-range energy functions. Moreover, Equation (6) is a particular case of Equation (15), when M=1 and $H_{\beta }$ is an energy function of range one.

The average values of the features, their correlations, as well as their higher cumulants can be obtained by taking the successive derivatives of the topological pressure with respect to their conjugate parameters β. This explains the important role played by the topological pressure in this framework. In general,

(16) $$\frac{\partial^{n}P\left[\;H_{\beta }\;\right]}{\partial \beta_{k}^{n}}=\kappa_{n}\;\forall k\in \left\{1,\ldots ,K\right\},$$

where $\kappa_{n}$ is the cumulant of order *n* (see Appendix B).

In particular, taking the first derivative:

(17) $$\frac{\partial P\left[\;H_{\beta }\;\right]}{\partial \beta_{k}}=E_{p}\left\{f_{k}\right\}=c_{k},\;\forall k\in \left\{1,\ldots ,K\right\},$$

where $E_{p}\left\{f\right\}$ is the the average of $f_{k}$ with respect to *p* (maximum entropy measure), which is equal (by assumption) to the average value of $f_{k}$ with respect to the empirical measure from the data $c_{k}$, that constraint of the maximization problem. These equations suggest a relationship with the logarithm of the free energy or log partition function of the Boltzmann–Gibbs distribution. Indeed, for range one potentials (time-independent Maximum entropy distributions), ρ(β)=Z(β) and $P\left[H_{\beta }\right]=\ln Z\left(\beta \right)$ which relates Equation (8) with Equation (17) (For a detailed example see Section 5.2). This problem of estimating the MEMC parameters become computationally expensive for big matrices. However, there exist efficient algorithms to estimate the parameters for the Markov maximum entropy problem in the literature [45].

## 4. Large Deviations and Applications in MEMC

### 4.1. Preliminary Considerations

This subsection reviews two elementary tools for studying the convergence of random variables while providing corresponding references. In the sequel, first the central limit theorem is introduced in Section 4.1.1, and then large deviation theory is discussed in Section 4.1.2.

#### 4.1.1. Central Limit Theorem

Let us first assume that one can have access to arbitrarily large data sequences. Consider t∈N and let $x_{0,t-1}$ be the spike-block of length *t* (which is allowed to increase), and let f:Ω→R be an arbitrary feature (not necessarily belonging to the set of features chosen to fit the MEMC). In this section, we establish asymptotic properties of $A_{t}\left(f\right)$ sampled with respect to the MEMC characterized by p{·}.

Throughout this work, we assume that p{·} is an ergodic Markov probability measure, meaning that every spiking block in $A_{R}^{N}$ is attainable from every other block in the Markov chain within *R* time steps as discussed in Section 3. Thanks to the ergodic assumption, it is guaranteed that the empirical averages become statistically more accurate as the sampling size grows [51], i.e.,

$$A_{t}\left(f\right)\to E_{p}\left\{f\right\}.$$

However, the above result does not clarify the rate at which the estimate accuracy improves. To answer this question, one can use the central limit theorem (CLT) for ergodic Markov chains (see [52]). This theorem states that there exists a constant σ>0 such that for large values of *t*, the random variable $\frac{\sqrt{t}}{\sigma }\left[A_{t}\left(f\right)-E\left\{f\right\}\right]$ distributes as a standard Gaussian random variable (technically, the central limit theorem says that

$$p\left\{\frac{\sqrt{t}}{\sigma }\left[A_{t}\left(f\right)-E\left\{f\right\}\right]\leq x\right\}\to \frac{1}{\sqrt{2\pi }\sigma }\int_{-\infty }^{x}e^{-\frac{s^{2}}{2\sigma }}ds,$$

where the convergence is in distribution), with σ being the square-root of the auto-covariance function of *f* [52]. This, in turn, implies that “typical” fluctuations of $A_{t}\left(f\right)$ around its mean value E{f} are of the order of $\sigma /\sqrt{t}$.

#### 4.1.2. Large Deviations

Although the central limit theorem for ergodic Markov chains is accurate in describing typical events (which are fluctuations around the mean value), it does not say anything about the likelihood of larger fluctuations. Even though it is clear that the probability of such large fluctuations goes to zero as the sample size increases, it is valuable to describe the corresponding decrease rate. In particular, one says that an empirical average $A_{t}\left(f\right)$ satisfies a large deviation principle (LDP) with rate function $I_{f}$, defined as

(18) $$I_{f}\left(s\right):=-\lim_{t\to \infty }\frac{1}{t}\log p\left\{A_{t}\left(f\right)>s\right\},$$

if the above limit exists. Intuitively, the above condition for large *t* implies that $p\left\{A_{t}\left(f\right)>s\right\}\approx e^{-tI_{f}\left(s\right)}$. In particular, if $s>E_{p}\left\{f\right\}$ the Law of Large Numbers (LLN) guarantees that $p\{A_{t}\left(f\right)>s\}$ tends to zero as *t* grows; the rate function quantifies the speed at which this happens.

Calculating $I_{f}$ directly, i.e., by using the definition (Equation (18)), can be a formidable task. However, the Gärtner–Ellis theorem provides a smart shortcut for avoiding this problem [27]. To this end, let us introduce the *scaled cumulant generating function* (SCGF) (the name comes from the fact that the *n*-th cumulant of *f* can be obtained by *t* successive differentiation operations over of $\lambda_{f}\left(k\right)$ with respect to *k*, and then evaluating the result at k=0) associated to the random variable *f*, by

(19) $$\lambda_{f}\left(k\right)=:\lim_{t\to \infty }\frac{1}{t}\ln E_{p}\left[e^{tkA_{t}\left(f\right)}\right],\;k\in R,$$

when the limit exists (further general details about cumulant generating functions are found in Appendix B). Note that, while $A_{t}\left(f\right)$ is an empirical average taken over a sample, the expectation in Equation (19) is taken over the probability distribution given by the corresponding model p{·}. If $\lambda_{f}$ is differentiable, then the Gärtner-Ellis theorem ensures that the average $A_{t}\left(f\right)$ satisfies a LDP with rate function given by the Legendre transform of $\lambda_{f}$, that is

(20) $$I_{f}\left(s\right)=\max_{k\in R}\left\{ks-\lambda_{f}\left(k\right)\right\}.$$

Therefore, in summary, one can study the large deviations of empirical averages $A_{t}\left(f\right)$ by first computing their SCGF from the selected model and then finding their Legendre transform.

One of the most useful applications of the LDP is to estimate the likelihood that $A_{t}\left(f\right)$ adopts a value far from its expected value. To illustrate this, let us assume that $I_{f}\left(s\right)$ is a positive differentiable convex function (A classical result in LDP states that $I_{f}\left(s\right)$ is a convex function if $\lambda_{f}\left(k\right)$ is differentiable [28]. For a discussion about the differentiability of $\lambda_{f}\left(k\right)$ see [53].). Then, because of the properties of convex functions $I_{f}\left(s\right)$ has a unique global minimum. Denoting this minimum by $s^{*}$, it follows from the differentiability of $I_{f}\left(s\right)$ that $I_{f}\left(s^{*}\right)=0$, and using properties of the Legendre transform $s^{*}=\lambda_{f}^{\prime }\left(0\right)=\lim_{t\to \infty }E_{p}\left(f\right)$. This is the LLN, i.e., $A_{t}\left(f\right)$ gets concentrated around $s^{*}$. Consider a value $s\neq s^{*}$ and assume that $I_{f}\left(s\right)$ admits a Taylor series around $s^{*}$ given by

$$I_{f}\left(s\right)=I_{f}\left(s^{*}\right)+I_{f}^{\prime }\left(s^{*}\right)\left(s-s^{*}\right)+\frac{I_{f}^{\prime \prime }\left(s^{*}\right){\left(s-s^{*}\right)}^{2}}{2}+O{\left(s-s^{*}\right)}^{3}$$

Since $s^{*}$ must correspond to a zero and a minimum of I(s), the first two terms in this series vanish, and as I(s) is convex function $I^{\prime \prime }\left(s\right)>0$. For large values of *k*, we obtain from Equation (18)

(21) $$\begin{array}{cc} p\{A_{t}\left(f\right)>s\} & \approx e^{-tI_{f}\left(s\right)}\\ & \approx e^{-t\left(\frac{I_{f}^{\prime \prime }\left(s^{*}\right){\left(s-s^{*}\right)}^{2}}{2}\right)} \end{array}$$

so the small deviations of $A_{t}\left(f\right)$ around $s^{*}$ are Gaussian-distributed as for i.i.d. sums $1/I_{f}^{\prime \prime }\left(s^{*}\right)=\lambda_{f}^{\prime \prime }\left(0\right)=\sigma^{2}$. In this sense, large deviation theory can be seen as an extension of the CLT because it gives information not only about the small deviations around $s^{*}$ but also about large deviations (not Gaussian) of $A_{t}\left(f\right)$.

### 4.2. Large Deviations for Features of MEMC

In this section, we focus on the statistical properties of features sampled from the inferred MEMC. For example, one may be interested in measuring the probability of obtaining “rare” average values of features such as firing rates, pairwise correlations, triplets or spatiotemporal events. This characterization is relevant as these features are likely to play an important role in neuronal information processing, and rare values may hinder the whole enterprise of conveying information. We show in this section how to obtain the large deviations rate functions of arbitrary features through the Gärtner–Ellis theorem via the SCGF. In particular, we show that the SCGF can be directly obtained from the inferred Markov transition matrix *P*.

Consider MEMC taking values on the state space $A_{R}^{N}$ with transition matrix *P*. Let *f* be a feature of range *R* which consider only the block and k∈R, we introduce ${\tilde{P}}^{\left(f\right)}\left(k\right)$, the *tilted transition matrix by f* of *P*, parameterized by *k*, whose elements are given by:

(22) $${\tilde{P}}_{ij}^{\left(f\right)}\left(k\right)=P_{ij}e^{kf(j)}\;i,j\in A_{R}^{N}.$$

For transition matrices *P* inferred from the MEP, the tilted transition matrix can be built directly from the spectral properties of the transfer matrix Equation (10) as follows,

(23) $$\begin{array}{ccc} {\tilde{P}}_{ij}^{\left(f\right)}\left(k\right) & = & \frac{e^{H_{\beta }\left(i,j\right)}V_{j}}{V_{i}\;\rho }e^{kf(j)}\\ & = & \frac{e^{\left[H_{\beta }\left(i,j\right)+kf\left(j\right)\right]}V_{j}}{V_{i}\;\rho }\;i,j\in A_{R}^{N}. \end{array}$$

Recall that *V* is the right eigenvector of the transfer matrix L. Here, we also have used the shortcut notation $H_{\beta }\left(i,j\right)$ to indicate that the energy function takes the contributions from the blocks *i* and *j*. Remarkably, the feature *f* does not need to belong to the set of chosen features to fit the MEMC.

Now, we can take advantage of the structure of the given process in order to obtain more explicit expressions for the SCGF $\lambda_{f}\left(k\right)$, for instance, if one considers i.i.d. random variables *X* then, from the very definition, one can obtain that

$$\lambda \left(k\right)=\lim_{t\to \infty }\frac{1}{t}\ln E{\left[e^{kX}\right]}^{t}=\ln E\left[e^{kX}\right],$$

which is the case of range one features. Thus, using Equation (22), we get that the maximum eigenvalue of the tilted matrix, denoted by $\rho ({\tilde{P}}_{f}\left(k\right))$ is,

$$\rho \left({\tilde{P}}_{f}\left(k\right)\right)=\sum_{j}\pi_{j}e^{kf(j)}\;j\in A_{1}^{N}.$$

Since ${\tilde{P}}_{f}$ is a positive matrix, the Perron–Frobenius theorem ensures the uniqueness of ρ .

Next, for the case of additive features, one deals with positive Markov chains, and under the assumption of ergodicity, an straightforward calculation (see, for instance, [54]) leads us to obtain that

(24) $$\lambda_{f}\left(k\right)=\ln\left(\rho \left({\tilde{P}}^{\left(f\right)}\right)\right).$$

**Figure 3:**
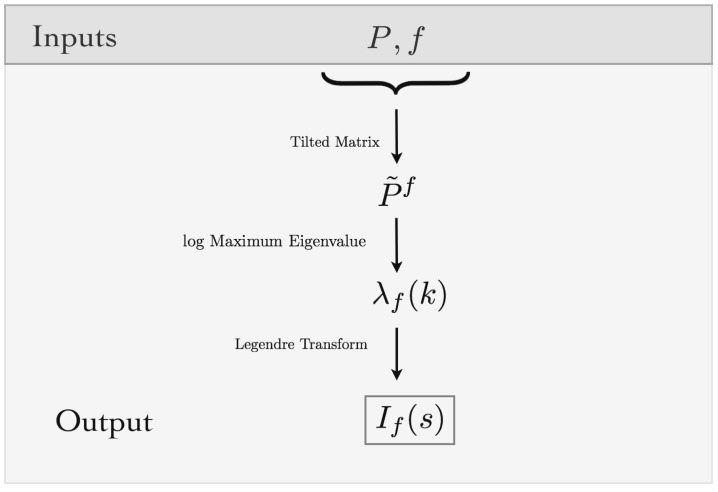
Algorithmic view of the method: Inputs are the maximum entropy Markov transition matrix and a feature. From the inputs, the tilted transition matrix can be built. The rate function of the feature is obtained as the Legendre transform of the log maximum eigenvalue of the tilted transition matrix. Using this framework, we can estimate the large deviations of the average values of the features.

It also can be proven that $\lambda_{f}\left(k\right)$, in this case, is differentiable [54], setting up the scene to apply the Gärtner–Ellis theorem, which bypasses the direct calculation of $p\{A_{T}\left(f\right)>s\}$ in Equation (18), i.e., having $\lambda_{f}\left(k\right)$, its Legendre transform leads to the rate function of *f* as shown in Figure 3.

### 4.3. Large Deviations for the Entropy Production

A stochastic process is said to be in equilibrium if one cannot notice the effect of time on it. It is worth noticing that non-equilibrium stochastic processes are natural candidates to model spike train statistics as time plays a non-symmetrical role [25]. One of the consequences of including features of range R>1 as constraints in the maximum entropy problem is that it opens the possibility to break the time-reversal symmetry present in the time-independent models. This captures the irreversible character of the underlying biological process and, thus, allows fitting more realistic statistical models from the biological point of view.

To characterize this mathematically, we study how the distribution p{·} changes when the time order is reversed. For this aim, let us consider a spike block $x_{0,T-1}=x_{0},x_{1},\ldots ,x_{T-1}$ containing *T* spike patterns, and define the time-reversed spike block $x_{0,T-1}^{\left(R\right)}$ obtained by re-ordering the time index in reverse order, i.e., $x_{0,T}^{\left(R\right)}=x_{T-1},x_{T-2},\ldots ,x_{2},x_{0}$.

A spiking network modeled by p{·} is said to be in equilibrium if $p\left\{x_{0,T}\right\}=p\left\{x_{0,T}^{\left(R\right)}\right\}$ for all x [55]. For a homogeneous discrete time ergodic Markov chain characterized by the Markov measure p(π,P) taking values in $A_{R}^{N}$, to be in equilibrium is equivalent to satisfy the *detailed balance conditions*, which is given by the following set of equalities:

$$\pi_{i}P_{ij}=\pi_{j}P_{ji},\;\forall i,j\in A_{R}^{N}.$$

Conversely, when these conditions are not satisfied, the statistical model of the spiking activity is said to be a non-equilibrium system. Since non-equilibrium is expected to occur generically in neuronal network models, one would like to quantify how far from equilibrium is the inferred MEMC. For this purpose, one can define the *information entropy production* (IEP) for *p*, which is given by

$$IEP\left(p\right):=\lim_{t\to \infty }\frac{1}{t}\ln\left[\frac{p\{x_{0,t-1}\}}{p\{x_{0,t-1}^{\left(R\right)}\}}\right],$$

when the limit exists. For the maximum entropy Markov measure p(π,P), the IEP is explicitly given by:

(25) $$IEP\left(\pi ,P\right)=\frac{1}{2}\sum_{i,j\in A_{R}^{N}}\left[\pi_{i}P_{ij}-\pi_{j}P_{ji}\right]\log\frac{\pi_{i}P_{ij}}{\pi_{j}P_{ji}},$$

(see [56] for the calculation). We remark that it is still possible to obtain the information entropy production rate also in the non-stationary case. Clearly, for features of range one, IEP=0 always, meaning that the process is time-reversible, therefore the probabilities of every path and its corresponding time-reversal path are equal. For features of range R>1, IEP>0 generically (we refer the interested reader to [25] for details and examples).

However, since in practice one only have access to limited amount of data, a natural question is to ask for the entropy production of the system considered up to a finite amount of time. It turns out that this characterization can be obtained through a LDP. With this in mind one may define the following feature:

$$W_{T}\left(x_{0,T-1}\right)=\frac{1}{T}\ln\left[\frac{p(x_{0,T-1})}{p(x_{0,T-1}^{\left(R\right)})}\right].$$

Since we have assumed that samples are produced by a stationary ergodic Markov chain characterized by p(π,P), the ergodic theorem assures that for *p*-almost every sample, the quantity $W_{t}$ when *t* goes to infinity converges, and it is by definition the IEP,

$$\lim_{t\to \infty }W_{t}\left(x_{0,t-1}\right))=IEP\left(\pi ,P\right).$$

Once we have the convergence for $W_{t}$, we may ask for its large deviation properties. Under the same idea above, and following [57], we introduce the following matrix:

$$F_{ij}=P_{ij}\ln{\left[\frac{\pi_{i}P_{ij}}{\pi_{j}P_{ji}}\right]}^{k}\;i,j\in A_{R}^{N},$$

this matrix help us to build the SCGF associated to $W_{t}$, through the logarithm of the maximum eigenvalue $\rho_{F}\left(k\right)$. Using the Gärtner–Ellis theorem one gets the rate function $I_{W}\left(s\right)$ for the IEP.

### 4.4. Large Deviations and MEMC Distinguishability

It is clear that there exist a relationship between accuracy of the estimation and sample size. The larger the sample size the more information is available and the uncertainty diminish. In the context of maximum entropy models, this idea has been well conceptualized using tools from information geometry [30,58]. The main idea of this approach is that the maximum entropy models form a manifold of probability measures whose coordinates are the parameters β. Consider a spike train dataset $x_{0,T-1}$ consisting of *T* spike patterns obtained from a spiking neuronal network. Given a set of features ${\left\{f_{k}\right\}}_{k=1}^{K}$ and their empirical averages, one may infer the parameters $\beta =(\beta_{1},\ldots ,\beta_{K})$ characterizing the MEMC p(π,P). We may use the inferred MEMC to generate a sample $x_{0,T-1}^{\prime }$ of the same size as the original dataset. Considering the same set of features one may apply again the MEP to infer a new set of parameters $\beta^{\prime }$ from $x_{0,T-1}^{\prime }$, which is, in general, expected to be different from β. These maximum entropy models will belong to a certain volume in the manifold which will decrease as the sample size increase [30]. On the other hand, increasing the sample size of $x_{0,T-1}^{\prime }$, one expects that the Markov chain $p^{\prime }\left(\pi^{\prime },P^{\prime }\right)$ specified by $\beta^{\prime }$ gets “closer” to the one characterized by β. The idea of relating a distance in the parameter space with a distance in the space of probability measures can be rigorously formulated using large deviations techniques. Let us start by defining the relative entropy between these two MEMC (Gibbs measures in the sense of Bowen in Equation (15)), which provides a notion of “distance” (the relative entropy is not a metric as is not symmetric and do not satisfy the triangle inequality). To do that in the context of MEMCs, consider a Gibbs measure *p* associated to the energy function $H_{\beta }$, and let *q* be another Gibbs measure. The Ruelle–Föllmer theorem gives us an expression for the relative entropy density between two Gibbs measures in terms of the pressure, the entropy rate and the expected value of the energy function with respect to *q* (see [29]), as follows:

(26) $$d\left(q|p\right)=P\left[H_{\beta }\right]-S\left(q\right)-E_{q}\left(H_{\beta }\right).$$

Observe that if d(q∣p)=0, we obtain the variational characterization of Gibbs measures in Equation (14).

Consider the potential $H_{\beta }=\sum_{k=1}^{K}\beta_{k}f_{k}$ associated with a MEMC p(π,P). Given a set of empirical averages $A_{t}\left(f_{k}\right)$ generated by a sample of p(π,P) we obtain new maximum entropy parameters $\beta^{\prime }$. The probability that the maximum entropy parameters $\beta^{\prime }$ associated with an ergodic Markov Chain $p^{\prime }\left(\pi^{\prime },P^{\prime }\right)$ get close to β follows the following large deviation principle [28]:

(27) $$\lim_{\delta \to 0}\lim_{t\to \infty }\frac{-1}{t}\ln P\left(|\beta -\beta^{\prime }|\in \Delta \delta \right)=d\left(p|p^{\prime }\right),$$

where $\Delta \delta ={\left[-\delta ,\delta \right]}^{K}$. Calling and the vector $\delta \beta =\beta -\beta^{\prime }$ and choosing Δδ close to 0, we informally rewrite the above corollary in the form:

(28) $$\frac{-1}{t}\ln P\left(|\delta \beta |\in \Delta \delta \right)\underset{t\to \infty }{⟶}d\left(p|p^{\prime }\right).$$

Thus, for large *T*, we get:

$$P\left(|\delta \beta |\in \Delta \delta \right)\approx e^{-td(p|p^{\prime })},$$

which implies that close-by parameters are associated to close-by probability measures [30].

Consider now two MEMCs p(π,P) and $p^{\prime }\left(\pi^{\prime },P^{\prime }\right)$ specified by $H_{\beta }$ and $H_{\beta^{\prime }}$, respectively with the same family of features. We say that the MEMCs are ϵ-*indistinguishable* if:

(29) $$-\ln P\left(|\delta \beta |\in \Delta \delta \right)\leq \epsilon .$$

As both MEMCs satisfy the variational principle (Equation (14)), the relative entropy between *p* and $p^{\prime }$ (Equation (26)) reads:

(30) $$d\left(p|p^{\prime }\right)=P\left[H_{\beta^{\prime }}\right]-P\left[H_{\beta }\right]+p\left(H_{\beta }\right)-p\left(H_{\beta^{\prime }}\right).$$

Taking the Taylor expansion of $d(p|p^{\prime })$ around $\beta^{\prime }=\beta$ we get:

$$d\left(p|p^{\prime }\right)\approx d\left(p|p\right)+\sum_{k}\frac{\partial d(p|p^{\prime })}{\partial \beta_{k}^{\prime }}{|}_{\begin{array}{c} \beta^{\prime }=\beta \end{array}}\left(\beta_{k}-\beta_{k}^{\prime }\right)+\frac{1}{2}\sum_{k,j}\frac{\partial^{2}d\left(p|p^{\prime }\right)}{\partial \beta_{k}^{\prime }\beta_{j}^{\prime }}{|}_{\begin{array}{c} \beta^{\prime }=\beta \end{array}}\left(\beta_{k}-\beta_{k}^{\prime }\right)\left(\beta_{j}-\beta_{j}^{\prime }\right).$$

Since $d(p|p^{\prime })$ is minimized at $\beta^{\prime }=\beta$, we obtain,

$$d\left(p|p^{\prime }\right)\approx \frac{1}{2}\sum_{k,j}\frac{\partial^{2}d\left(p|p^{\prime }\right)}{\partial \beta_{k}^{\prime }\beta_{j}^{\prime }}{|}_{\begin{array}{c} \beta^{\prime }=\beta \end{array}}\left(\beta_{k}-\beta_{k}^{\prime }\right)\left(\beta_{j}-\beta_{j}^{\prime }\right).$$

Taking the second derivative of $d(p|p^{\prime })$ from (Equation (30)), one also has that,

(31) $$\frac{\partial^{2}d\left(p|p^{\prime }\right)}{\partial \beta_{k}^{\prime }\beta_{j}^{\prime }}=\frac{\partial^{2}P\left[H_{\beta^{\prime }}\right]}{\partial \beta_{k}^{\prime }\beta_{j}^{\prime }}=L_{kj}.$$

The second partial derivatives of the topological pressure with respect to the parameters $\beta_{k}^{\prime }$ and $\beta_{j}^{\prime }$ can be conveniently arranged in a matrix *L* with components $L_{kj}$. Given two MEMCs specified by $H_{\beta }$ and $H_{\beta^{\prime }}$, in the limit of large *t* they are ϵ-indistinguishable if:

(32) $$\frac{1}{2}\left[{\left(\delta \beta \right)}^{T}L\left(\delta \beta \right)\right]\leq \frac{\epsilon }{T},$$

where T denotes transpose. The matrix *L* can be obtained from data without need to fit the parameters. Equation (32) characterize a region in the space of MEMC of indistinguishable models, whose volume can be calculated in the large *t* limit using spectral properties of the matrix *L* [30]. This result generalizes a previous result for maximum entropy distributions for range one energy functions in [31].

## 5. Illustrative Examples

In this section, we illustrate the presented methods in some simple scenarios. In these examples, we follow a set of steps:

1. Choose the features and build the energy function (Equation (7)).
2. Build the transfer matrix (Equation (10)).
3. Compute the free energy and find the maximum entropy parameters using (Equation (17)).
4. Build the Markov transition matrix using (Equation (12)).
5. Choose the observable to examine and build the tilted transition matrix using Equation (22).
6. Compute the Legendre transform of the log maximum eigenvalue of the tilted transition matrix to obtain the rate function (Equation (24)).

For the sake of clarity, in this section, we focus on small neuronal networks. It is clear, however, that the extension of these techniques to larger neural populations is straightforward.

### 5.1. First Example: Maximum Entropy Model of a Range Two Feature

Consider spiking data from two interacting neurons. We measure only the average value of a of a range two feature from the spiking data to fit a MEMC. The feature denoted by $f_{1}$ is given by ${\tilde{f}}_{1}\left(x_{0,1}\right)=x_{0}^{2}\cdot x_{1}^{1}$, which detects when a spike of the second neuron is followed by a spike in the first one. The system can be described with the help of an energy function $H\left(x_{0,1}\right)=\beta_{1}{\tilde{f}}_{1}\left(x_{0,1}\right)$.

For a given dataset of *T* spike blocks of range two, the empirical average reads,

(33) $$A_{T}\left(f_{1}\right)=c$$

This means that in the data one finds that this event appears c% of the time.

The transfer matrix $L_{H}$ (cf. Equation (10)) is primitive by construction (cf. Equation (10)) and satisfies the hypothesis of the Perron–Frobenius theorem. In fact, its unique maximum eigenvalue is $\rho \left(\beta_{1}\right)=e^{\beta_{1}}+3$. Given the restriction in Equation (33), using Equation (17) we obtain the following relationship between the parameter $\beta_{1}$ and the value of the restriction *c*:

$$\frac{\partial P\left[\;H\;\right]}{\partial \beta_{1}}=\frac{\partial \log(e^{\beta_{1}}+3)}{\partial \beta_{1}}=\frac{e^{\beta_{1}}}{e^{\beta_{1}}+3}=c.$$

**Figure 4:**
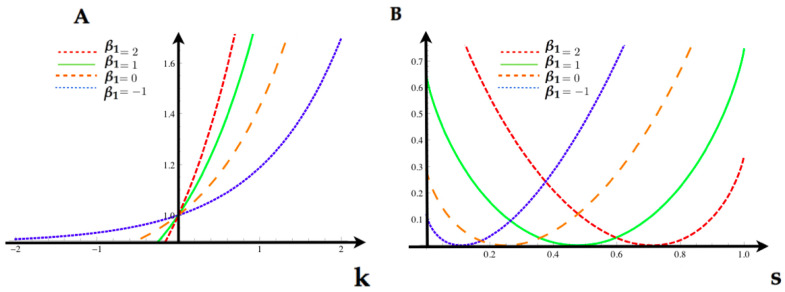
(**A**) Scaled cumulant generating function (SCGF) (Equation (24)) for the feature $f_{1}$ of the first example computed at the values provided by the table above. (**B**) Rate function for the same feature computed at the same parameter values as the SCGF. Each of this functions are obtained taking the Lagrange transform of the respective SCGF in (**A**).

This equation can be solved numerically. Using the obtained value of $\beta_{1}$ in Equation (12), one can find the corresponding Markov transition matrix. Note that, among all the Markov chains that match exactly the restriction, the selected one maximizes the KSE. Moreover, it is direct to check that the variational principle in Equation (14) is satisfied. Examples of values of $\beta_{1}$ for different values of *c* and IEP (25) for each value of $\beta_{1}$ are given in the following table:

Having the MEMC, we are now interested in analyzing the statistical fluctuations of the feature $f_{1}$. Using Equation (22), we obtain the tilted transition matrix ${\tilde{P}}_{ij}^{\left(f_{1}\right)}\left(k\right)$ for each of the values in the Table 1. In Figure 4, we compute for each value of $\beta_{1}$ we compute the SCGF $\lambda_{f_{1}}\left(k\right)$ (24) and the Legendre transform (rate function) associated to the feature $I_{f_{1}}\left(s\right)$.

**Figure 5:**
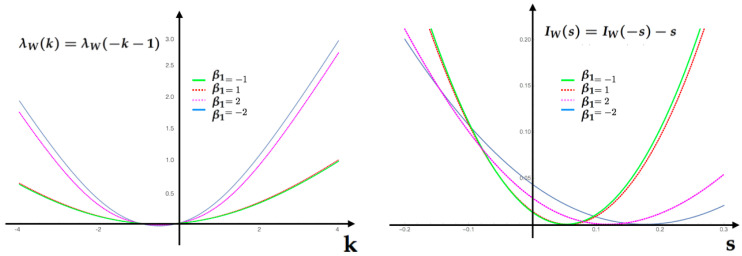
Gallavotti–Cohen symmetry relationship for the information entropy production IEP for values in Table 1. Left SCGF $\lambda_{W}\left(k\right)$. Right rate function of the IEP feature $W,I_{W}\left(s\right)$.

In Figure 5, we compute for each value of IEP in the table the rate function and illustrate for this example the symmetry relationship (Equation (A9)).

### 5.2. Second Example: Maximum Entropy Model With Only Synchronous Constraints

Let us now consider a network of three neurons. We focus here on range one features. In this example, we consider features related to the firing rates and synchronous pairwise correlations (Ising model [6,7]). Specifically, we consider the following energy function:

$$H\left(x_{0}\right)=\beta_{1}x_{0}^{1}+\beta_{2}x_{0}^{2}+\beta_{3}x_{0}^{3}+\beta_{4}x_{0}^{1}\cdot x_{0}^{2}+\beta_{5}x_{0}^{1}\cdot x_{0}^{3}+\beta_{6}x_{0}^{2}\cdot x_{0}^{3},$$

with the six parameters $\beta_{1},\ldots ,\beta_{6}$. Following (Equation (10)), the transfer matrix $L_{H}$ indexed by the states of $A_{1}^{3}$ is the following:

$$L_{H}=\left(\begin{array}{cccccccc} 1 & e^{\beta_{1}} & e^{\beta_{2}} & e^{\beta_{1}+\beta_{2}+\beta_{4}} & e^{\beta_{3}} & e^{\beta_{1}+\beta_{3}+\beta_{5}} & e^{\beta_{2}+\beta_{3}+\beta_{6}} & e^{\beta_{1}+\beta_{2}+\beta_{3}+\beta_{4}+\beta_{5}+\beta_{6}}\\ \vdots & \vdots & \vdots & \vdots & \vdots & \vdots & \vdots & \vdots \\ 1 & e^{\beta_{1}} & e^{\beta_{2}} & e^{\beta_{1}+\beta_{2}+\beta_{4}} & e^{\beta_{3}} & e^{\beta_{1}+\beta_{3}+\beta_{5}} & e^{\beta_{2}+\beta_{3}+\beta_{6}} & e^{\beta_{1}+\beta_{2}+\beta_{3}+\beta_{4}+\beta_{5}+\beta_{6}} \end{array}\right).$$

This matrix is primitive, and the unique maximum eigenvalue is

$$\rho \left(\beta \right)=1+e^{\beta_{1}}+e^{\beta_{2}}+e^{\beta_{1}+\beta_{2}+\beta_{4}}+e^{\beta_{3}}+e^{\beta_{1}+\beta_{3}+\beta_{5}}+e^{\beta_{2}+\beta_{3}+\beta_{6}}+e^{\beta_{1}+\beta_{2}+\beta_{3}+\beta_{4}+\beta_{5}+\beta_{6}}.$$

The right eigenvector associated to this eigenvalue has all the components equal to 1. We obtain the topological pressure $P\left[\;H\;\right]=\log\rho \left(\beta \right)$. To find the MEMC parameters, we solve this set of equations:

(34) $$\frac{\partial P\left[\;H\;\right]}{\partial \beta_{1}}=A_{T}\left(f_{k}\right)=c_{k}.$$

From Equation (34), provided some constraints on the average value of the features, we can solve the maximum entropy problem (see Table 2).

**Figure 6:**
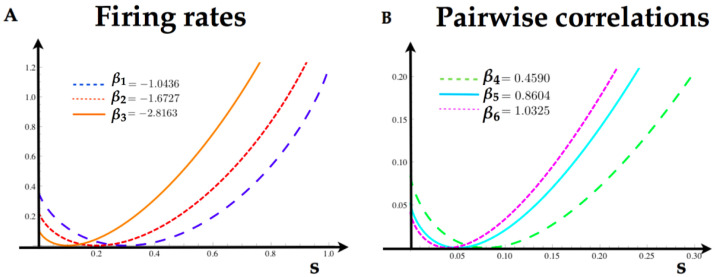
(**A**) Rate functions for the firing rates of each neuron of the Ising model. The minimum of the rate functions coincide with the expected value of the firing rates in Table 2. (**B**) Rate functions for the pairwise interactions computed from the parameters in Table 2.

From Equation (12), one can find the Markov transition matrix. To compute the rate function of each feature in this model, we take the logarithm of the maximum eigenvalue of the tilted matrix, and obtain the tilted cumulant generating function $\lambda_{f}\left(k\right)$. In Figure 6, we illustrate the rate functions for each feature in the model.

### 5.3. Third Example: Past Independent and Markov Maximum Entropy Measures

To illustrate the difference between synchronous and non-synchronous maximum entropy models, we study a simple model composed of two interacting neurons:

(35) $$H\left(x_{0,1}\right)=\beta_{1}x_{0}^{1}\cdot x_{1}^{2}+\beta_{2}x_{0}^{2}\cdot x_{1}^{1}+\beta_{3}x_{0}^{1}\cdot x_{0}^{2}.$$

We build a Markov chain by fixing the parameters of H at $\beta_{1}=-3,\beta_{2}=3,\beta_{3}=0.5$ in the state space $A_{1}^{2}$, given by

$$\left\{\left(\;\begin{array}{c} 0\\ 0 \end{array}\;\right),\left(\;\begin{array}{c} 0\\ 1 \end{array}\;\right),\left(\;\begin{array}{c} 1\\ 0 \end{array}\;\right),\left(\;\begin{array}{c} 1\\ 1 \end{array}\;\right)\right\}.$$

whose corresponding transition matrix is given by

$$P=\left(\begin{array}{cccc} 0.13026 & 0.02580 & 0.65762 & 0.18632\\ 0.65763 & 0.13026 & 0.16529 & 0.04682\\ 0.02580 & 0.10266 & 0.13026 & 0.74128\\ 0.15015 & 0.59735 & 0.03774 & 0.21476 \end{array}\right).$$

We focus on the synchronous feature $f=x_{0}^{1}\cdot x_{0}^{2}$, whose average value with respect to the Markov measure *p* fixed by the parameters $\beta_{1},\beta_{2},\beta_{3}$ is $E_{p}\left\{x_{0}^{1}\cdot x_{0}^{2}\right\}=0.292611$.

Using this particular Markov chain, we generate a sample of size T=20,000. Then, we consider these data as a spike train of two neurons from which we have no other information. Starting from this data, we find the maximum entropy distribution that only considers the empirical average of the synchronous feature $A_{T}\left(x_{0}^{1}\cdot x_{0}^{2}\right)=0.2926$ as constraint. Therefore, we build a second model that uses the following energy function:

(36) $$\tilde{H}\left(x_{0}\right)=\tilde{\beta }x_{0}^{1}\cdot x_{0}^{2}.$$

Using the constraint, we obtain from Equation (8), $\tilde{\beta }=0.215874$ fixing the maximum entropy distribution $\tilde{p}$. Note that by construction $E_{\tilde{p}}\left\{x_{0}^{1}\cdot x_{0}^{2}\right\}=A_{T}\left(x_{0}^{1}\cdot x_{0}^{2}\right)=0.2926$.

**Figure 7:**
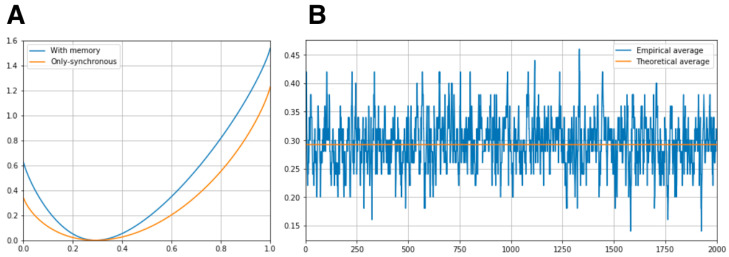
(**A**) Rate functions of the synchronous feature $x_{0}^{1}x_{0}^{2}$ for both energy functions. (**B**) Moving averages computed from a sample of length 20,000 of the Markov Chain with transition matrix *P*.

For both energy functions (Equations (35) and (36)) with the parameters mentioned before, we compute the rate functions of the synchronous feature. Additionally, from the sample of the Markov chain we compute the empirical averages of the synchronous feature using sliding windows of 50 samples. As expected, these empirical averages fluctuate around the overall average, as shown in Figure 7.

**Figure 8:**
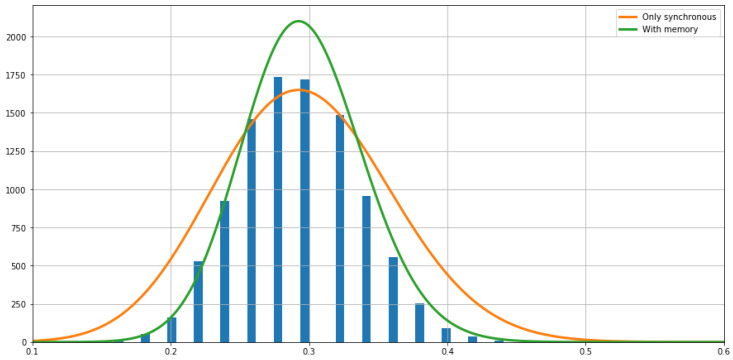
The fluctuations of the synchronous feature around the mean computed from the sample of the Markov chain are indicated with the bars. The Gaussian fluctuations around the mean predicted by the large deviations rate functions of both models are plotted. The curve predicted by the Markov model obtained from H is in green and the curve predicted by the model obtained from $\tilde{H}$ is in orange.

To test the relevance of including memory into the model (and assess the performance of memoryless features), we compared the statistics of the fluctuations seen in the empirical average with the prediction by the rate functions of the two models. Figure 8 shows the histogram of empirical fluctuations, and plots the theoretical estimations of the fluctuations given by $K\exp\{-WI_{f}\left(s\right)\}$, where W=50 is the window size, *s* is the fluctuation size, $I_{f}\left(s\right)$ is the rate function, and *K* is a constant that is adapted for visualization purposes.

Results show that the rate function of the model with memory fits accurately the relative frequencies of the empirical large fluctuations. In contrast, the model with no memory overestimates the fluctuation frequencies, having a much larger variance than the data, and underestimates near the expected value.

## 6. Conclusions

In the past few years, new experimental techniques combined with clever ideas from statistical mechanics have made it possible to infer maximum entropy models of spike trains directly from experimental recordings. However, a very important issue, namely quantifying the accuracy of the estimation obtained from a finite empirical sample, is usually ignored in this field. This is probably because the maximum entropy approach has a dual nature; one side is a convex optimization problem, which provides a unique solution independent of the sampling size, and on the other side is a Bayesian inference procedure, from which it is more natural to ask this question. As we have discussed in the Section 1 this characterization is relevant in the field of computational neuroscience as, in practice, experimental recordings are performed during a finite amount of time which causes fluctuations over the estimated quantities.

A fundamental goal of spike train analysis over networks of sensory neurons involves building accurate statistical models that predict the response of the network to a stimulus of interest. In particular, the aim of statistical inference of spiking neurons using the MEP, is that the fitted parameters shed light on some aspects of the neuronal code, therefore it is extremely important to quantify the accuracy of the statistical procedure. Additionally, one may be interested in measuring some properties of the inferred statistical model characterizing the spiking neuronal network, for example, the convergence rate of a sample or to quantify the probability of rare events of features such as firing rates, pairwise correlations, triplets or spatiotemporal events, mainly because these features are likely to play an important role in neuronal information processing. It is possible that rare and unlikely events have been generated by internal states of the neuronal tissue and not driven by the external stimulus. The events that are unlikely to occur deserve a better understanding as may carry important information about the network internal structure and may play a role in organizing a coherent dynamic to convey sensory information to the cerebral cortex.

The present contribution addressed this issue using tools from large deviations theory in the context of the MEMC. In particular, we showed that the transfer matrix technique used to build the MEMC is well adapted to compute large deviation rate functions using the Gärtner–Ellis theorem. We also provide tools to investigate how sharply determined are the parameters of a MEMC with respect to the amount of empirical data using the concept of ϵ distinguishability. Additionally, we present a non-trivial relation between the distance in the parameter space and the distance in the manifold of maximum entropy probability measures using a LDP.

We have illustrated our method using simple examples. However, these examples might give a false impression that large deviations rate functions can always be calculated explicitly. In fact, exact and explicit expressions can be found only in small simple cases; fortunately, there exist numerical methods to evaluate rate functions [53].

Here, we have focused our attention on large deviations properties on maximum entropy models arising from spike train statistics, however, these results can be used in other fields of applications of maximum entropy models.

## Figures and Tables

**Table 1:** Set of values used in Figure 4 and Figure 5.

*c*	$\beta_{1}$	IEP
0.043	−2	0.176
0.11	−1	0.056
0.25	0	0
0.475	1	0.0525
0.711	2	0.1184

**Table 2:** Set of values used in Figure 6.

$A_{T}\left(f_{k}\right)$	$c_{k}$	$\beta_{k}$	$\delta \beta_{k}$	${\tilde{c}}_{k}$
$A_{T}\left(x^{1}\right)$	0.3	−1.0436	0	0.30350016
$A_{T}\left(x^{2}\right)$	0.2	−1.6727	0	0.20127414
$A_{T}\left(x^{3}\right)$	0.1	−2.8163	0	0.10450018
$A_{T}\left(x^{1}x^{2}\right)$	0.08	0.4590	0	0.08187418
$A_{T}\left(x^{1}x^{3}\right)$	0.05	0.8604	0.1	0.05475019
$A_{T}\left(x^{2}x^{3}\right)$	0.04	1.0325	0	0.04207419

## Abbreviations

The following abbreviations are used in this manuscript:

MEP	Maximum entropy principle
MEMC	Maximum entropy Markov chain
SCGF	Scaled cumulant generating function
CLT	Central limit theorem
LLN	Law of large numbers
LDP	Large deviation principle
IEP	Information entropy production
KSE	Kolmogorov–Sinai entropy
NESS	Non-equilibrium steady states

**Symbol list**

$x_{n}^{k}$	Spiking state of neuron *k* at time *n*.
$x_{n}$	Spike pattern at time *n*
$x_{t_{1},t_{2}}$	Spike block from time $t_{1}$ to $t_{2}$.
$A_{T}\left(f\right)$	Empirical Average value of the feature *f* considering *T* spike patterns.
$A_{R}^{N}$	Set of spike blocks of *N* neurons and length *R*.
$S\left[\;p\;\right]$	Entropy of the probability measure *p*.
H	Energy function.
$P\left[\;H\;\right]$	Free energy or topological pressure.
$\lambda_{f}\left(k\right)$	Scaled cumulant generating function of *f*.
$I_{f}\left(s\right)$	Rate function of *f*.

## Appendix A. Discrete-Time Markov Chains and Spike Train Statistics

Consider the random process $\left\{X_{n}:n\geq 0\right\}$ taking values on $A_{R}^{N}$. One can assume that the spiking activity of the neuronal network can be modeled by some discrete-time Markov process whose transition probabilities are obtained by means of the maximum entropy method described in Section 3. In this setting, $A_{R}^{N}$ is the state space of the Markov chain, and, thus, if $X_{n}=x_{n,n+R-1}$, we say that the process is in the state $x_{n,n+R-1}$ at time *n*. The transition probabilities are given as follows,

(A1) $$P\left[X_{n}=x_{\left(n\right)}|X_{n-1}=x_{\left(n-1\right)},\ldots ,X_{0}=x_{\left(0\right)}\right]=P\left[X_{n}=x_{\left(n\right)}|X_{n-1}=x_{\left(n-1\right)}\right],$$

where we used the short hand notation $x_{\left(n\right)}:=x_{n,n+R-1}$. We emphasize that in this paper the states are spike blocks of finite length *R*, $x_{n,n+R-1}$. All along this paper he have only considered homogeneous Markov chains, that is, Equation (A1) is independent of *n*.

Since transitions are considered between blocks of the form $x_{n-R,n-1}\to x_{n-R+1,n}$, the block $x_{n-R+1,n-1}$ must be common for the transition to be possible. Consider two spike blocks $i,j\in A_{R}^{N}$ of range R≥2. We say that the transition from state *i* to state *j* is *allowed* if *i* and *j* have the common sub-block $x_{1,R-1}={\tilde{x}}_{0,R-2}$, where ${\tilde{x}}_{0,R-2}$ are the first R−1 spike patterns of *j*.

Now, we define the transition matrix $P_{R}:A_{R}^{N}\times A_{R}^{N}\to R$, whose entries are given by the transition probabilities, as follows,

(A2) $${\left(P_{R}\right)}_{ij}:=\left\{\begin{array}{cc} P\left[j|i\right]>0\; & \mathrm{if}\;i\to j\;\mathrm{is}\;\mathrm{allowed}\\ 0,\; & \mathrm{otherwise}. \end{array}\right.$$

Note that *P* has $2^{NR}\times 2^{NR}$ entries, but it is a sparse matrix since each line has, at most, $2^{N}$ non-zero entries. A stochastic matrix *P* is defined from transition probabilities in Equation (A2) satisfying:

$$P\left[j|i\right]\geq 0;\;\;\sum_{j\in A_{R}^{N}}P\left[j|i\right]=1,$$

for all states $i,j\in A_{R}^{N}$. Moreover, by construction, for any pair of states, there exists a path of maximum length *R* in the graph of transition probabilities going from one to the other, which means that the Markov chain is primitive.

## Appendix B. Cumulant Generating Function

In general, to obtain the scale cumulant generating function (as considered in Section 4.1.2), one has to deal with the moment of order *r* of a real-valued random variable *f*, which is given by,

$$m_{r}=E\left(f^{r}\right),$$

for r∈N. Provided that it has a Taylor expansion about the origin, the moment generating function (or Fourier-Laplace transform)

$$M\left(k\right)=E\left(e^{kf}\right)=E\left(1+kf+\cdots +k^{r}f^{r}/r!+\cdots \right)=\sum_{r=0}^{\infty }m_{r}k^{r}/r!$$

The cumulants $\kappa_{r}$ are the coefficients in the Taylor expansion of the cumulant generating function, defined as the logarithm of the moment generating function, that is,

$$\log M\left(k\right)=\sum_{r}\kappa_{r}k^{r}/r!$$

The relationship between the first moments and cumulants can be obtained by extracting coefficients from the expansion, as follows:

$$\begin{array}{ccc} \kappa_{1} & = & m_{1}\\ \kappa_{2} & = & m_{2}-m_{1}^{2}\\ \kappa_{3} & = & m_{3}-3m_{2}m_{1}+2m_{1}^{3}\\ \kappa_{4} & = & m_{4}-4m_{3}m_{1}-3m_{2}^{2}+12m_{2}m_{1}^{2}-6m_{1}^{4}, \end{array}$$

and so on. In particular, $\kappa_{1}$ is the mean of *f*, $\kappa_{2}$ is the variance, $\kappa_{3}$ the skewness and $\kappa_{4}$ the kurtosis.

## Appendix C. Linear Response

Within the framework we have built, we can quantify how a small perturbation of the maximum entropy parameters (associated to given features) affects the average values of other features of the MEMC. It is important to quantify this perturbation because the maximum entropy parameters are obtained with finite accuracy due to finite sample effects. Fixing β, we can obtain the average value of a given feature $f_{k}$ with respect to the MEMC without need to sample, using the Gibbs–Jaynes principle for the KSE [29], which asserts that for a translation invariant probability measure *p*, the entropy rate $S_{KS}\left(p\right)$ is maximal under the constraints $E_{p}\left\{f_{k}\right\}=c_{k}$, for all k∈{1,…,K} if and only if *p* is a Gibbs measure associated to the energy $H_{\beta }=\sum \beta_{k}f_{k}$, where $E_{p}\left\{f_{k}\right\}=\frac{\partial P[H_{\beta }]}{\partial \beta_{k}}=c_{k}$.

Now, let us consider a perturbed version of the energy denoted by $H_{\beta +\delta \beta }$. Using a Taylor expansion, we compute the average value of an arbitrary feature here denoted by $f_{k}$ with respect to the MEMC associated to the perturbed energy in terms of the unperturbed one, that is,

(A3)Epβ+δβ{fk}=∂PHβ+δββk(A4)=∂PHββk+∑j∂2PHβ∂βkβjδβj+O(δβj)2(A5)=Epβ{fk}+∑j∂2PHβ∂βkβjδβj+O(δβj)2=c˜k.

From Equations (A3) and (A4), there is a Taylor expansion of $P\left[\;H_{\beta +\delta \beta }\;\right]$ about $H_{\beta }$. From Equations (A4) and (A5), we use the Gibbs–Jaynes principle for the KSE. We see from Equation (A5) that a small perturbation of a parameter $\beta_{j}$ influence the average value of all other features in the energy function (as $f_{k}$ is arbitrary) and the magnitude of the perturbation is controlled by the second derivatives of the topological pressure of the unperturbed energy $P\left[\;H_{\beta }\;\right]$.

## Appendix D. Time Correlations from Topological Pressure

For a pair of finite range features $f_{k},f_{j}$ of a stationary Markov chain, the covariance of order *r* is independent of time, only depends on the lag *r* and is defined as:

$$C_{f_{k},f_{j}}\left(r\right):=E_{p}\left\{f_{k}\left(x_{n}\right)f_{j}\left(x_{n+r}\right)\right\}-E_{p}\left\{f_{k}\left(x_{n}\right)\right\}E_{p}\left\{f_{j}\left(x_{n}\right)\right\},$$

where $E_{p}$ stands for the expected value with respect to the Markov measure *p*.

For MEMC with potentials of range R>1, there is a positive time correlation correlation between pairs of features $f(x_{n})$ and $g(x_{n+r})$, which we denote $\sigma_{f,g}^{2}$; indeed, one can show that (Green–Kubo formula):

(A6) $$\sigma_{f_{k},f_{j}}^{2}=C_{f_{k},f_{j}}\left(0\right)+\sum_{r=1}^{\infty }C_{f_{k},f_{j}}\left(r\right)+\sum_{r=1}^{\infty }C_{f_{j},f_{k}}\left(r\right).$$

The pairwise time correlations between features can be obtained from the topological pressure:

(A7) $$\sigma_{f_{k},f_{j}}^{2}=\frac{\partial^{2}P\left[H_{\beta }\right]}{\partial \beta_{k}\;\partial \beta_{j}}=\frac{\partial \mu \left[\;f_{j}\;\right]}{\partial \beta_{k}}.$$

for a MEMC taking values on $A_{R}^{N}$ characterized by p(π,P) :

$$\frac{\partial^{2}P\left[H_{\beta }\right]}{\partial \beta_{k}\;\partial \beta_{j}}=E_{p}\left\{f_{k}f_{j}\right\}-E_{p}\left\{f_{k}\right\}E_{p}\left\{f_{j}\right\}+\sum_{r=1}^{\infty }\sum_{y,w\in A_{R}^{N}}f_{k}\left(y\right)f_{j}\left(w\right)\pi_{y}P_{yw}^{r}+\sum_{r=1}^{\infty }\sum_{y,w\in A_{R}^{N}}f_{j}\left(y\right)f_{k}\left(w\right)\pi_{y}P_{yw}^{r}$$

for $v,w\in A_{R}^{N}$. For MEMC fitted through range one energy functions, $\left\{f\left(x_{n}\right);n\geq 0\right\}$ is an i.i.d. process and the variance of *f* is simply $C_{f}\left(0\right)$. These terms are the linear response coefficients. For MEMC associated to energy functions formed by *K* features, the matrix *L* can be conveniently arranged in a K×K symmetric matrix (known as the Onsager reciprocity relations [59]).

(A8) $$\sigma_{f_{k},f_{j}}^{2}=\frac{\partial^{2}P\left[H_{\beta }\right]}{\partial \beta_{k}\;\partial \beta_{j}}=\frac{\partial E_{p}\left\{f_{j}\right\}}{\partial \beta_{k}}.$$

## Appendix E. Gallavotti–Cohen Fluctuation Theorem

The Gallavotti–Cohen fluctuation theorem refers to a universal property of the IEP, i.e., it is independent of the parameters of the MEMC. It is as a statement about properties of the SCGF and rate function of the IEP [57], this is,

(A9) $$\lambda_{W}\left(k\right)=\lambda_{W}\left(-k-1\right),\;I_{W}\left(s\right)=I_{W}\left(-s\right)-s.$$

This symmetry can be seen as a generalization of Kubo formula in Equation (A6) and Onsager reciprocity relations in Equation (A8) to situations far from equilibrium. It is a relationship that holds for a general class fs stochastic processes including Markov chains [55].

These properties have an impact on the large deviations of the time-averaged entropy production rate of the sample trajectory $x_{0,t-1}$ of the Markov chain p(π,P) denoted $\frac{W_{t}}{t}$. In our framework, the following relationship always holds,

$$\frac{p\left\{\frac{W_{t}}{t}\approx s\right\}}{p\left\{\frac{W_{t}}{t}\approx -s\right\}}≍e^{ts}.$$

This means that the positive fluctuations of $\frac{W_{t}}{t}$ are exponentially more probable than negative fluctuations of equal magnitude.
